# The evaluation of an artificial intelligence system for estrus detection in sows

**DOI:** 10.1186/s40813-023-00303-3

**Published:** 2023-03-15

**Authors:** Steven Verhoeven, Ilias Chantziaras, Elise Bernaerdt, Michel Loicq, Ludo Verhoeven, Dominiek Maes

**Affiliations:** 1 Unit of Porcine Health Management, Department of Internal Medicine, Reproduction and Population Medicine, Faculty of Veterinary Medicine, Ghent University, Salisburylaan 133, 9820 Merelbeke, Belgium; 2 Department of Internal Medicine, Reproduction and Population Medicine, Faculty of Veterinary Medicine, Ghent University, Salisburylaan 133, 9820 Merelbeke, Belgium; 3 noHow, Sluizenstraat 119, 8450 Bredene, Belgium; 4 Eindhoven, The Netherlands; 5 Present Address: Lintjeshof, Pannenweg 200, 6031 RK Nederweert, The Netherlands

**Keywords:** Pig production, Estrus detection, Artificial intelligence, Reproductive performance

## Abstract

**Background:**

Good estrus detection in sows is essential to predict the best moment of insemination. Nowadays, a technological innovation is available that detects the estrus of the sow via connected sensors and cameras. The collected data are subsequently analyzed by an artificial intelligence (AI) system. This study investigated whether such an AI system could support the farmer in optimizing the moment of insemination and reproductive performance.

**M&M:**

Three Belgian sow farms (A, B and C) where the AI system was installed, participated in the study. The reproductive cycles (n = 6717) of 1.5 years before and 1.5 years after implementation of the system were included. Parameters included: (1) farrowing rate (FR), (2) percentage of repeat-breeders (RB), (3) farrowing rate after first insemination (FRFI) and (4) number of total born piglets per litter (NTBP). Also, data collected by the system were analyzed to describe the weaning-to-estrus interval (WEI), estrus duration (ED) and the number of inseminations used per estrus. This dataset included 2261 cycles, collected on farms B and C.

**Results:**

In farm A, all parameters significantly improved namely FR + 4.3%, RB − 3.75%, FRFI + 6.2% and NTBP + 1.06 piglets. In farm B, the NTBP significantly decreased with 0.48 piglets, but in this farm the insemination dose was too low (0.8 × 10^9^ spermatozoa per dose). In farm C, only the NTBP significantly increased with 0.45 piglets after the implementation of the system. The WEI as determined by the system varied between 78 and 90 h, being 10–20 h shorter in comparison with the WEI as determined by the farmer. The ED, determined by the system ranged from 48 to 60 h, and was less variable as compared to the ED as assessed by the farmer. The mean number of inseminations per estrus remained similar over time in farm B whereas it decreased over time from approximately 1.6–1.2 in farm C.

**Conclusion:**

The AI system can help farmers to improve the reproductive performance, assess estrus characteristics and reduce the number of inseminations per estrus. Results may vary between farms as many other variables such as farm management, genetics and insemination dose also influence reproductive performance.

## Background

Several studies emphasize that proper estrus detection in sows is essential to predict the best moment for insemination and to obtain optimal reproductive performance [1–3]. In order to achieve optimal conception rates and minimize regular repeat breeders, it is necessary that insemination is done properly and at the right moment [4, 5]. The latter is also important to achieve a high number of total and live born piglets [6]. Ideally, insemination should be performed 0–24 h before ovulation [4, 5, 7]. However, in commercial farm circumstances it is hard to determine the moment of ovulation. In general, the estrus duration (ED) varies between 24 and 96 h with an average of 40–60 h. Previous studies have shown that ovulation occurs when 70% of the ED has passed [8]. Steverink et al. [2] showed that the ED and the weaning-to-estrus interval (WEI) are inversely related to each other. In other words, the shorter the WEI, the longer the ED and the longer it will take before ovulation takes place. An increase of the WEI from 3 to 6 days was associated with a decrease of the ED from 55 to 37 h [5]. It has also been shown that ED in gilts and repeat breeders is shorter than in sows with higher parities [2, 6].

Since the reproductive performance highly depends on the time of insemination relative to ovulation, a good monitoring of the onset of the estrus is critical to determine the best moment of insemination. In commercial breeding farms, estrus detection is often done visually by the farmer, based on the behavior of the sow and externally assessable physiological changes. Farm workers commonly use the characteristic standing estrus reflex to determine estrus. Other sow behavioral signs include nervousness, growling, tipping of the ears and loss of appetite. External assessable signs are mucosal vaginal discharge and a swollen, reddish colored vulva [8]. However, these signs are highly variable between sows and ED is hardly predictable in advance. Therefore, multiple inseminations are applied per estrus to optimize fertility results. This strategy is time consuming and incurs extra costs because of the multiple doses of sperm that are used per estrus.

Technological innovations using connected sensors and cameras that continuously monitor behavioral data have been developed for estrus detection in the sow. The data are subsequently analyzed by an artificial intelligence (AI) system to provide estrus characteristics and support the farmer in determining the best moment of insemination [9, 10]. Therefore, these techniques may help the farmer in facilitating estrus detection and insemination strategies, and improve reproductive performance.

The present study investigated whether a real-time AI system for estrus detection in sows based on monitoring the sow behavior can support pig farmers to determine the best moment of insemination and to improve reproductive performance. Three Belgian commercial farms where the system was installed were included. The WEI, the ED, and the number of applied artificial inseminations per estrus were also investigated.

## Materials and methods

### Artificial intelligence system for estrus detection in sows

The system that was used in the present is called the SmaRt Sow Breeding (SSB) (Conception Ro-main Inc., Québec Canada), formerly called PigWatch [9, 10]. This system continuously collects behavioral data of each sow in the breeding unit via a camera that is fixated on the crate above the sow. These data are sent to a data analysis cabinet where the data are processed. The activity patterns of the sows are displayed as a graph on the interface that can be consulted by the farmer. Based on these patterns, the algorithm makes a prediction for the best moment of insemination for each individual sow and an insemination request is displayed on the user’s interface.

The system has been designed to be used in weaned sows, not in gilts as their behavior is too variable and difficult to assess reliably. Every individual sow shows unique activity and behavior patterns. The first 2 days after weaning are used by the system to learn the unique behavior of each individual sow prior to estrus onset and to use them as a baseline. After this period, the system assesses significant changes in behavior patterns which are characteristic for the estrus of each individual sow. These data are used to predict the ideal moment of insemination.

The system also relies on input given by the farmer next to the data that it collects by itself. Farmers using the system are recommended to perform estrus detection with a teaser boar once per day and to indicate the time when they perform estrus detection. Also, farmers should feed the sows maximum twice per day and at fixed times, allowing the system to discriminate between behavior related to feeding and behavior related to estrus. Finally, it should be as quiet as possible in the insemination unit in order to minimize the risk of sows showing any irregular behavior that is not related to estrus, and make it easier for the system to detect signals of estrus.

Both the onset as well as the ending of the estrus, characterized by the standing estrus reflex, needs to be registered in the system by the farmer. This additional information is used by the algorithm in the following ways [10].

- At the time the system asks for an insemination request, the algorithm validates whether the farmer already detected estrus for this individual sow. If the farmer already detected estrus, the system asks for insemination and it will assume that this is the best moment for breeding. All insemination requests are done autonomically by the system. If the farmer did not confirm estrus yet, the algorithm will redo the calculation to verify whether the farmer missed an estrus. Only in cases of disagreement between the system and the farmer, the system will switch to a manual mode.
- If the system detects a malfunction of the sensors such as a dirty sensor collecting unreliable data, the system will request insemination during the entire estrus period as determined by the farmer.
- If the farmer determines the sow to be in estrus during the first 2 days post weaning, the system will automatically ask for a daily insemination request during the observed ED since the system did not have enough time to analyze the sow’s activity patterns in anestrus.
- If the algorithm detects an erratic pattern of activity, it will automatically ask for one insemination per day for the duration of the estrus as determined by the farmer.
- If the system found the sow in estrus some time ago but no ideal moment of insemination was detected, the system will automatically ask for an insemination in order to avoid a missed estrus.

Some weaned sows should ideally be inseminated during the night. As most farms do not have a night shift to perform these inseminations, the system requests a preventive insemination just before the end of the workday. If some of these sows do not have their best moment to breed overnight, the system asks for a second insemination in the morning or later in order to be closer to the ideal moment of insemination.

### Reproductive performance data

Three Belgian farms where the system was installed and that were willing to participate were included in the study. The main characteristics of the farms are given in Table 1.

**Table 1:** General characteristics of each farm included in this study

	Farm A	Farm B	Farm C
Herd size (number of sows)	250	450	300
Sow batch production system	5	4	3
Weaning age (days)	24	21	24
Origin of the breeding gilts	Own breeding	Purchase	Own breeding
Breed of the sows	English landrace × large white	TN70	TN70
Dose of sperm applied (number of spermatozoa)	2.4 × 10^9^	0.8 × 10^9^	1.6 × 10^9^

Within each farm, the reproductive data of a period of 1.5 year before implementation of the system was compared with those of 1.5 year after implementation of the system. In total, 6,717 cycles of weaned sows were analyzed. Table 2 shows the distribution of cycles for the three farms.

**Table 2:** Number of sow reproductive cycles included in the study in the three farms (A, B and C). Data were collected 1.5 years before and 1.5 years after using the SmaRt Sow Breeding (SSB) system (N = 6717)

Farm	Start SSB	Number of sow reproductive cycles before/after using SSB		Total cycles
		Before	After	
A	24-OCT-2016	863	871	1734
B	10-OCT-2018	1515	1527	3042
C	23-SEP-2019	976	965	1941

Upon using the SSB system, the herd management generally remained the same, except for the type of pipettes used for insemination and the insemination dose. Prior to implementation of the system, all farms used classical pipettes for cervical or post-cervical insemination. Upon implementation of the SBB, and according to the recommendations of the manufacturer, special pipettes allowing intra-uterine insemination were used. Insemination with these pipettes can be done without the need to have the presence of a teaser boar. The manufacturer of the system also advised to use at least 1.6 × 10^9^ spermatozoa per dose. Prior to the study, the doses contained approximately 2.4 × 10^9^ spermatozoa, which is higher than the amount advised for conventional use (4) (11). In this sense, it was indicated to the farmers that lowering the dose to two thirds of the original dose was allowed. This was done in Farm C. However, farm A preferred to continue using the original dose. Farm B had misunderstood the recommendation and lowered the insemination dose to one third of the original dose namely 0.8 × 10^9^. He thought that lowering of the dose to one third was allowed, instead of lowering the dose by one third. This became clear only after the study was performed. As farm B adhered to all other aspects of the protocol, including assessing estrus characteristics (see further), the data of farm B were maintained in the study.

The sow reproductive data were collected from the management software of the farms. Farm A used a commercial software program (Pig’Up, ISAGRI France), whereas farms B and C used another program (Ceres, AgroVision Belgium). All the data was exported to a standardized dataset prior to analysis in order to streamline all the results.

For the three farms, four reproductive performance parameters were analyzed, namely: (1) farrowing rate (FR), (2) percentage of repeat-breeders (RB), (3) farrowing rate after first insemination (FRFI) and (4) number of total born piglets per litter (NTBP). Since gilts were not monitored by the system, their results were not included. FR was obtained by dividing the total number of sows that farrowed by the total number of sows that was inseminated. The percentage of RB was determined by dividing the number of sows that were rebred by the total number of sows that had been inseminated. The FRFI was determined by dividing the total number of sows that farrowed minus the sows that were rebred, by the total number of sows that were inseminated. The NTBP included liveborn, stillborn and mummified piglets.

### Weaning-to-estrus interval, estrus duration and number of inseminations

The parameters weaning-to-estrus interval (WEI), estrus duration (ED), and number of inseminations were measured or calculated after implementation of the AI system. Thus, regarding those analyses, only data in the new situation was available and only data that was available at the beginning of this study was used. As mentioned earlier, the system relies on a daily estrus detection and stimulation by the farmer. Therefore, the farmer had to record the onset and end of the estrus in the system. The system also registered its findings for both variables. Based on the onset and the end of the estrus, WEI and ED as determined by the farmer and the system was calculated.

The data on ED was only available for farm B and C. Farm A did not have reliable registration of the data and was therefore excluded from this analysis. Farm B had data available from the onset of implementation of the SSB until September 2020, covering a total period of almost 2 years. Data for farm C included more or less 16 months, from the onset of using the system until January 2021. In some cases, it was recognized that not all variables were present in all records. For example, in some cases the system or the farmer did not register the beginning or end of the estrus, making an incomplete record. Only complete records, i.e. with accurate data of both the system and the farmer, were used to analyze the estrus characteristics. Records with missing data were therefore deleted. In total, 2261 cycles were analyzed.

The number of inseminations per estrus were also recorded for farm B and C.

### Data analysis

The statistical analyses regarding the reproductive performance data were processed in Minitab Workspace 18®. FR, RB and FRFI were all analyzed per individual herd, by doing a two-proportion t-test. The NTBP was first checked for normality by performing a normal probability plot. If normally distributed, these variables were tested by a two-sample t-test. A *p* value of ≤ 0.05 was considered significant. The WEI, ED and the number of inseminations were analyzed using a mixed model including the effects of weaning batch (random factor), season and parity (fixed factors). Those analyses were done in IBM® SPSS® Statistics for Windows Version 24 (IBM Corp., Armonk, N.Y., USA).

## Results

### Reproductive performance data

The results of the FR, RB, FRFI and NTBP before and after using the system on each farm are shown in Tables 3, 4, 5 and 6, respectively. In farm A, all four variables significantly improved after using the AI system namely FR + 4.3%, RB − 3.75%, FRFI + 6.2% and NTBP + 1.06 piglets. In farm B, only the difference in NTBP was statistically significant, i.e. − 0.48 piglets. FR and FRFI improved, whereas the RB increased (*p* > 0.05). In farm C, only NTBP significantly improved with 0.45 piglets. FR and FRFI improved as well, whereas RB increased (*p* > 0.05).

**Table 3:** Farrowing rate (FR) of sows (n = 6,717) before and after using the SmaRt Sow Breeding (SSB) system in farms A, B and C

	FR (%)		Difference (%)	*p* value	95% CI
	Before	After			
A	82.9	87.1	+ 4.3	0.015^*^	[0.8, 7.5]
B	82.4	82.6	+ 0.2	0.882	[− 2.5, 2.9]
C	86.0	88.6	+ 2.6	0.093	[− 0.4, 5.5]

*The *p* value was considered statistically significant (*p* ≤ 0.05)

**Table 4:** Percentage of repeat breeders (RB) in sows (n = 6,717) before and after using the SmaRt Sow Breeding (SSB) system in farms A, B and C

	RB (%)		Difference (%)	*p* value	95% CI
	Before	After			
A	7.76	4.01	− 3.75	0.001^*^	[− 5.9, − 1.5]
B	7.19	8.78	+ 1.58	0.108	[− 0.3, 3.5]
C	7.78	9.84	+ 2.06	0.110	[− 0.5, 4.6]

*The *p* value was considered statistically significant (*p* ≤ 0.05)

**Table 5:** Percentage of sows (n = 6717) that farrowed after first insemination (FRFI) before and after using the SmaRt Sow Breeding (SSB) system in farms A, B and C

	FRFI(%)		Difference (%)	*p* value	95% CI
	Before	After			
A	77.3	83.5	+ 6.2	0.001^*^	[2.5, 9.9]
B	76.2	76.6	+ 0.4	0.771	[− 2.6, 3.4]
C	80.6	81.0	+ 0.4	0.822	[− 3.1, *.9]

*The *p* value was considered statistically significant (*p* ≤ 0.05)

**Table 6:** Number of total born piglets per litter (NTBP) of sows (n = 6717) before and after using the SmaRt Sow Breeding (SSB) system in farms A, B and C

	NTBP		Difference	*p* value	95% CI
	Before	After			
A	13.96	15.02	+ 1.06	< 0.001^*^	[0.669, 1.449]
B	16.47	15.98	− 0.48	0.002^*^	[− 0.784, − 0.183]
C	15.29	15.74	+ 0.45	0.010^*^	[0.107, 0.796]

*The *p* value was considered statistically significant (*p* ≤ 0.05)

### Weaning-to-estrus interval, estrus duration and number of inseminations

The next results include only analyses of farms B and C since farm A did not have a proper registration of all data, as mentioned in the materials and methods.

#### Weaning-to-estrus interval

**Fig. 1:**
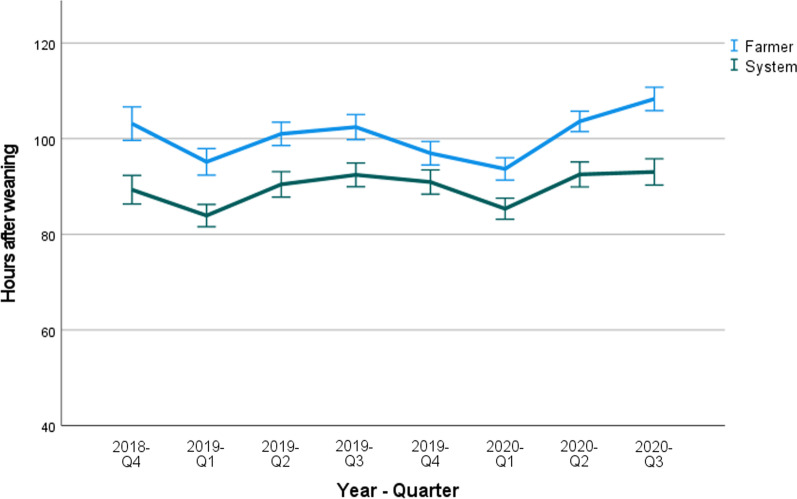
Farm B: Mean onset of the estrus after weaning (hours), shown per quarter (Q1–Q4) of the year as determined by the farmer and the SmaRt Sow Breeding (SSB) system. Error bars show 95% confidence interval

**Fig. 2:**
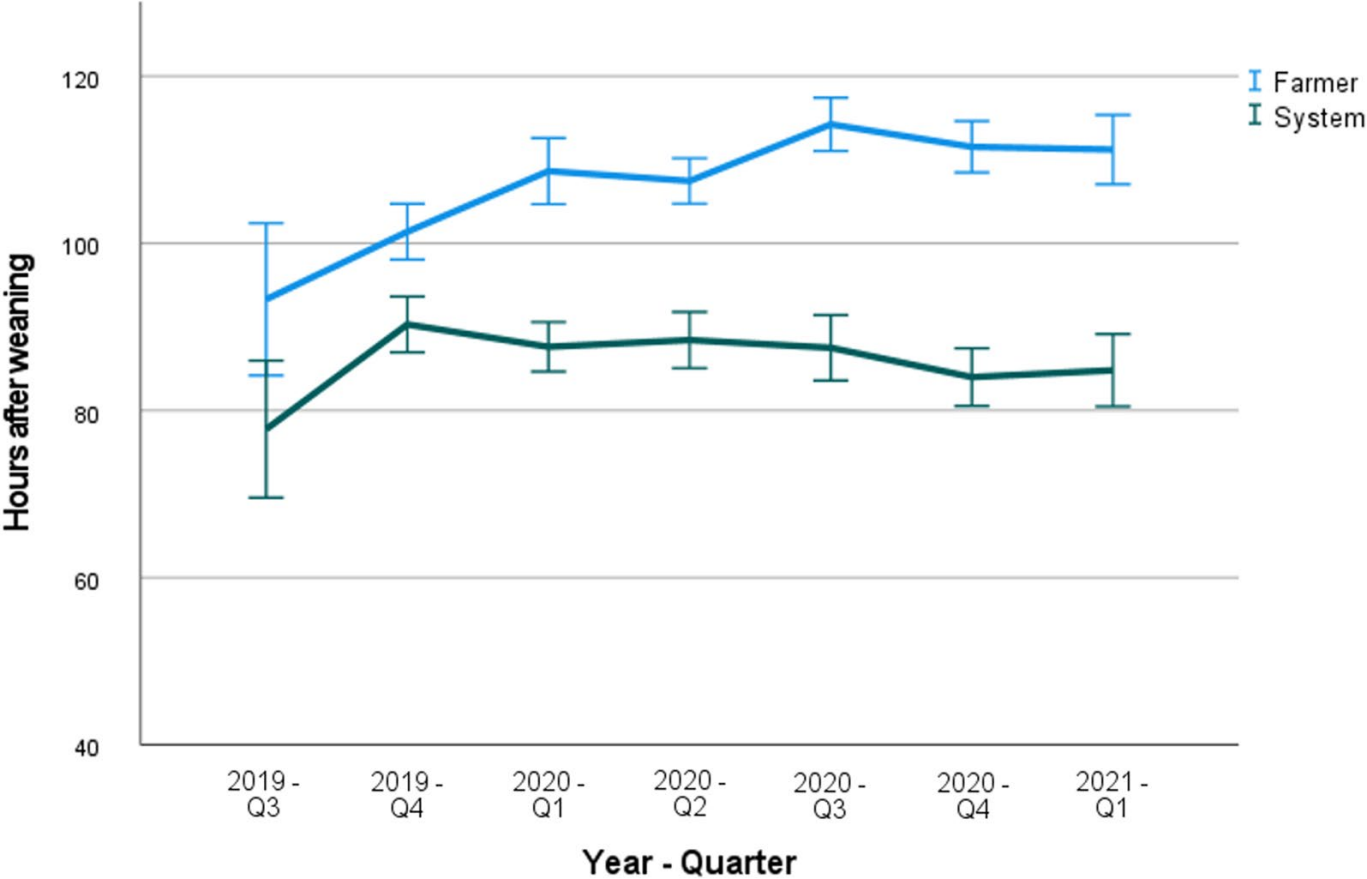
Farm C: Mean onset of the estrus after weaning (hours), shown per quarter (Q1–Q4) of the year as determined by the farmer and the SmaRt Sow Breeding (SSB) system. Error bars show 95% confidence interval

The WEI on farms B and C in each quarter of the year after implementation of the system are shown in Figs. 1 and 2, respectively. The onset of the estrus as determined by the system in farm B varied between 82 and 90 h after weaning. The farmer determined this onset later, varying between 95 and 108 h after weaning. In farm C, the system determined the onset of the estrus on average between 78 and 89 h after weaning, the farmer between 92 and 112 h after weaning on average.

The relationship between WEI and parity is shown in Table 7. When looking at differences between parities at farm B (*p* < 0.001), we found that the WEI in sows with parity 3 or 4 was 92.75 h, and 92.42 h for sows with parity 5 or higher. Younger sows with parity 1 and 2 came in estrus later, 99.94 and 98.00 h after weaning, respectively. In farm C, no significant differences in WEI were observed between parities (*p* = 0.490).

**Table 7:** Estimated means of weaning-to-estrus interval (WEI)^a^, estrus duration (ED)^a^ and number of inseminations per estrus according to parity in farms B and C, as determined by the system. Variables in the same row with different superscript are significantly different

	Parity				*p* value
	1	2	3–4	5+	
*Farm B*					
WEI (hours)	99.94^a^	98.00^a^	92.75^b^	92.42^b^	< 0.001^*^
ED (hours)	62.21^a^	64.15^a,b^	65.48^b^	65.31^a,b^	0.036^*^
Number of inseminations	1.70^a^	1.56^b^	1.57^b^	1.50^b^	< 0.001^*^
*Farm C*					
WEI (hours)	98.13	97.97	96.19	97.65	0.490
ED (hours)	56.42^a^	52.76^a,b^	50.36^b^	51.15^b,c^	0.001^*^
Number of inseminations	1.63^a^	1.46^b^	1.28^c^	1.30^b,c^	< 0.001^*^

*The *p* value was considered statistically significant (*p* ≤ 0.05)

#### Estrus duration

**Fig. 3:**
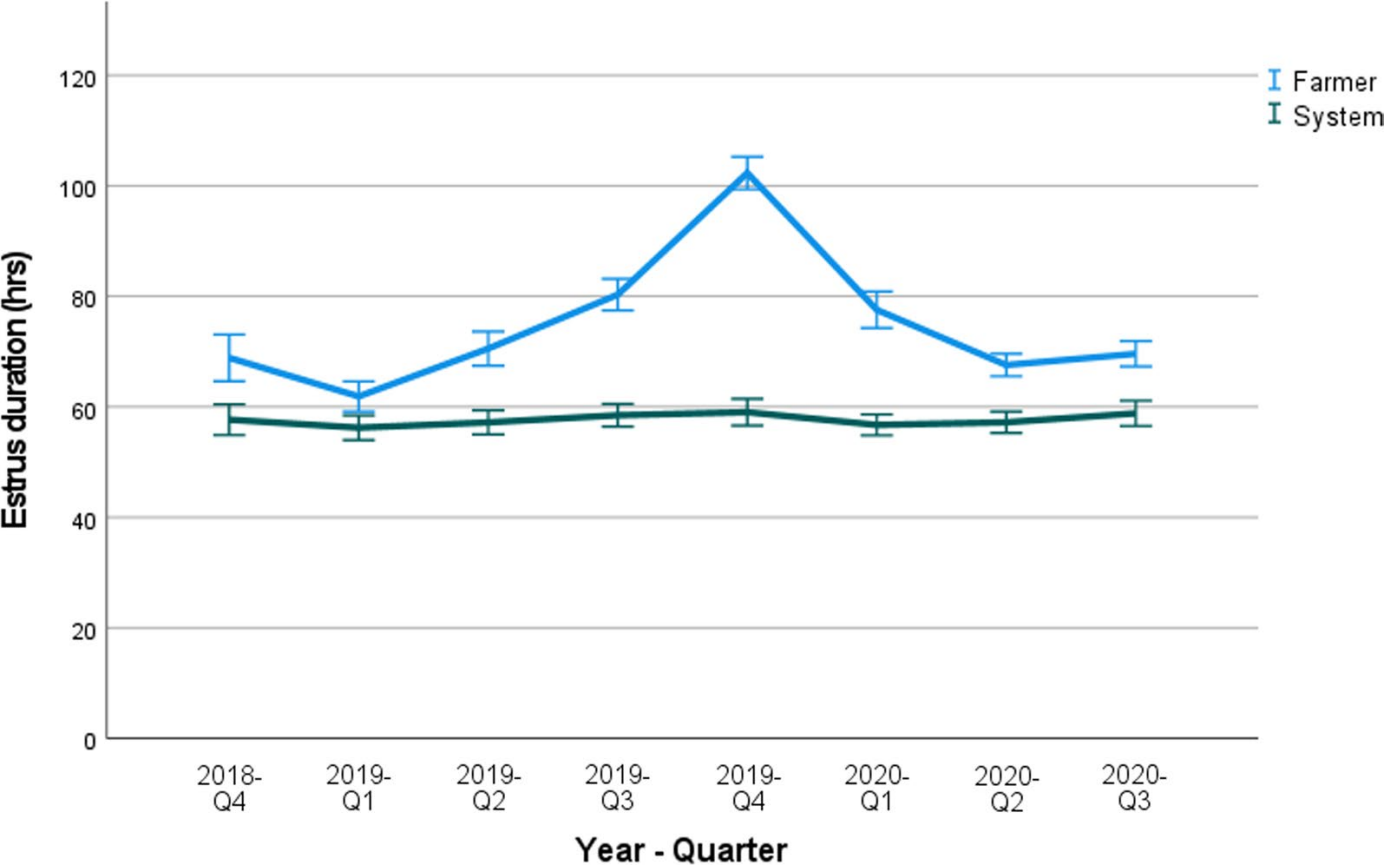
Farm B: Mean estrus duration (hours), shown per quarter (Q1–Q4) of the year as determined by the farmer and the SmaRt Sow Breeding (SSB) system. Error bars show 95% confidence interval

**Fig. 4:**
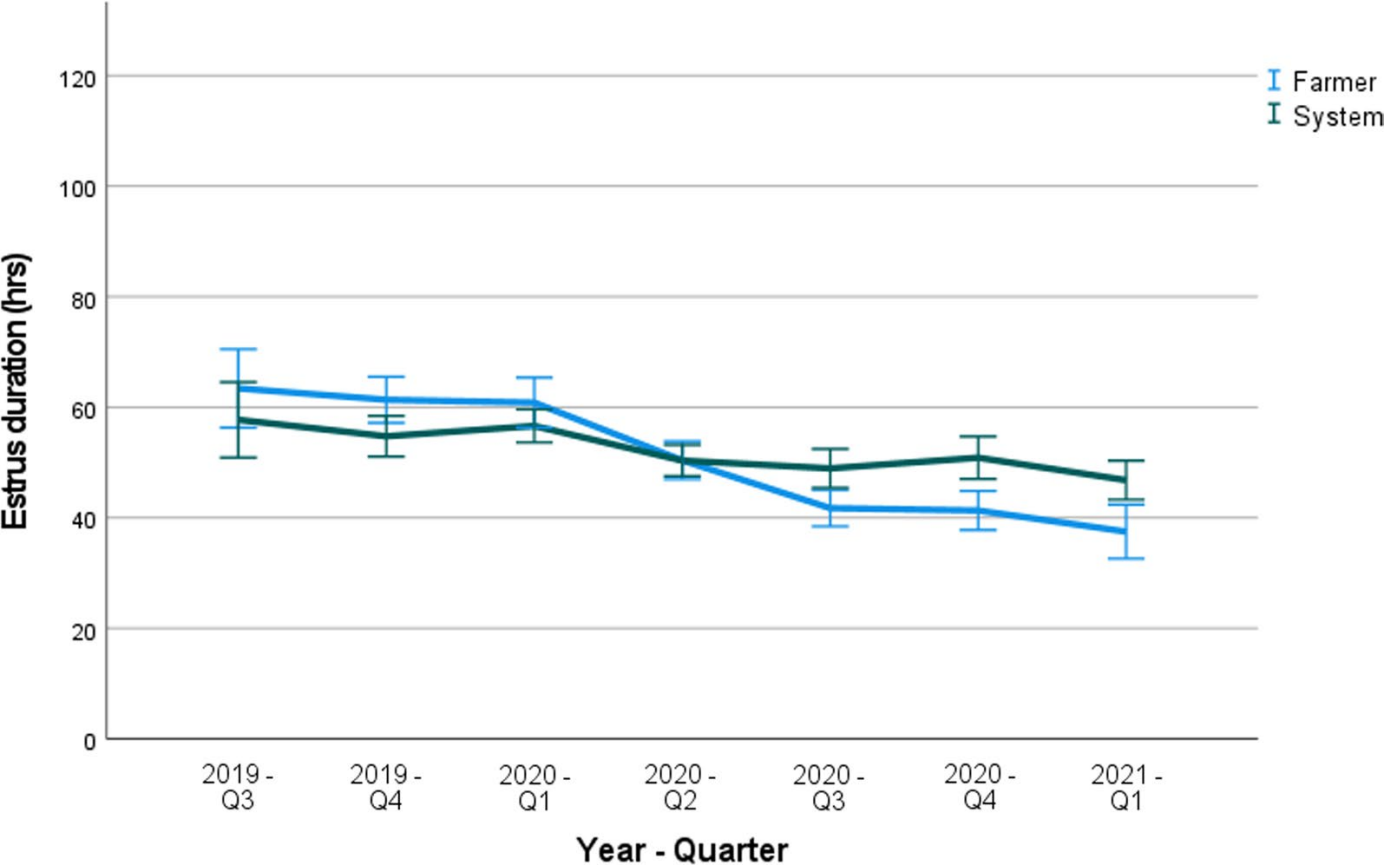
Farm C: Mean estrus duration (hours) as determined by the farmer and the SmaRt Sow Breeding (SSB) system, shown per quarter (Q1–Q4) of the year. Error bars show 95% confidence interval

The ED on farms B and C in each quarter of the year are shown in Figs. 3 and 4, respectively. The average ED in farm B measured by the system varied from 58 to 60 h, measured by the farmer between 62 and 101 h. In farm C, the ED determined by the system and the farmer was 48–57 and 38–63 h, respectively.

The results of the ED according parity are shown in Table 7. In farm B, the ED in parity 1 sows (62 h) was significantly shorter than the ED in parity 3 and 4 sows (65 h) (*p* = 0.036). In farm C, the ED was significantly longer in parity 1 sows (56 h) than in older sows (parity 3–4: 50 h, parity 5 or higher: 51 h) (*p* < 0.001).

#### Number of inseminations per estrus

**Fig. 5:**
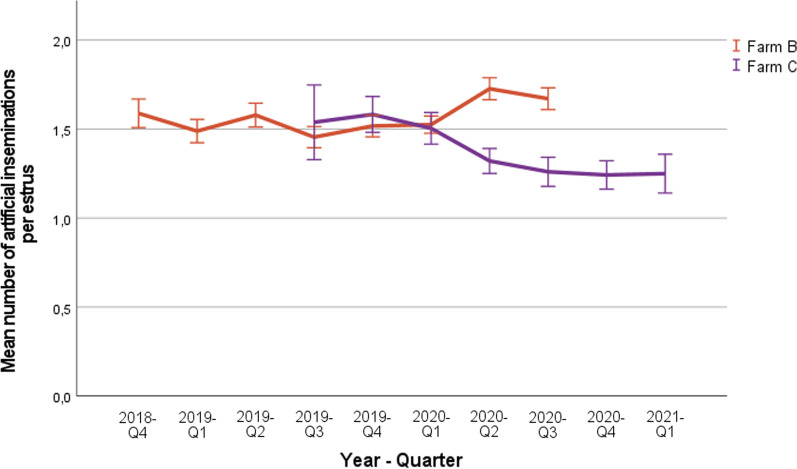
Mean number of artificial inseminations applied per estrus, expressed per quarter of the year in farms B and C after using the SmaRt Sow Breeding (SSB) system. Error bars show 95% confidence interval.

Figure 5 shows the mean number of inseminations that were used per estrus on each farm, for each quarter of the year. In farm B, values varied between 1.51 and 1.63 inseminations per estrus. In farm C, values ranged between 1.57 and 1.25, and showed a decreasing trend when the system was used for a longer time.

The number of inseminations applied per estrus according to the parity of the sows are shown in Table 7. On farm B, there were significantly more inseminations per estrus in parity 1 sows (1.70) compared to older sows (parity 2: 1.56; parity 3–4: 1.57; parity 5 or higher: 1.50, respectively (*p* < 0.001). This was also the case on farm C (*p* < 0.001). The number of inseminations per estrus in farm C were as follows: parity 1: 1.63; parity 2: 1.46; parity 3–4: 1.28 and parity 5 or higher 1.30. The difference between sows with parity 2 and parity 3–4 in farm C was also significant (*p* < 0.001).

## Discussion

This study showed that a real-time artificial intelligence system for estrus detection in sows can assist farmers to determine the best moment of insemination and if used properly, improve reproductive performance of the farm. Also, such a system is useful to easily determine the WEI and ED in a farm, and it may decrease the number of inseminations per estrus.

The overall results on reproductive performance were positive, but the study also showed that the results vary per farm. In farm A, where the original semen dose for insemination of 2.4 × 10^9^ spermatozoa was maintained, all reproductive performance parameters significantly improved namely FR + 4.3%, RB − 3.75%, FRFI + 6.2% and NTBP + 1.06 piglets. Farm C had lowered the semen dose with one third, namely from 2.4 × 10^9^ to 1.6 × 10^9^ spermatozoa per insemination, according to the recommendations of the manufacturer of the SSB system. In this farm, the litter size significantly increased with 0.45 piglets, but the other parameters did not statistically change after the implementation of the SSB system. There were only numeric (*p* > 0.05) improvements of FR and FRFI, and a numeric increase of the percentage of RB. However, in farm B, no significant changes were observed for FR, the percentage of RB and FRFI, but the NTBP decreased significantly with 0.48 piglets. However, due to a misunderstanding, this farm had used a too low number of spermatozoa for insemination namely 0.8 × 10^9^ spermatozoa per dose, which is less than half of an advised dose in conventual insemination [4, 11]. In this sense, the results on reproductive performance in this farm cannot be considered as a result of the implementation of the system. The results of farm B were maintained as for all other aspects of the study, the farm had fully adhered to the study protocol and was very cooperative. The results of WEI and ED were considered not to be affected by the insemination dose. It also emphasizes the importance of a very good communication between the manufacturer and the farmer when implementing the SSB. Finally, despite the fact that a too low insemination dose was used, the reproductive performance data were still high and more or less similar as before the system was introduced. That was likely also one of the reasons why the low semen dosage used became clear only after completing the study, else it would have become clear at an earlier stage. This illustrates that apart from semen dosage, also other management procedures such as a correct timing of insemination are very important. [4, 6, 12–14]

In farms B and C, data on WEI, ED and the number of inseminations were collected. In both farms, the WEI determined by the system was approximately 10–20 h shorter in comparison with the WEI as determined by the farmer. This implies that the first part of the estrus might often be missed by the farmers.

The ED in both farms as determined by the AI system ranged from 50 to 60 h. This is in agreement with ED durations mentioned in literature, where values ranging from 40 to 60 h have been reported [3]. In farm B, the ED as assessed by the farmer was similar, but consistently higher than the ED measured by the SSB. However, in farm C the ED as determined by the farmer was higher during the first three quarters of the study, and then lower during the remaining quarters of the study. When looking at the data in Figs. 3 and 4, the ED as assessed with the system was less variable within and also between quarters of the study compared to the ED as assessed by the farmer. The mean ED assessed by the farmer in quarter 4 of 2019 in farm B (Fig. 3) was approximately 20 h longer than during the other quarters of the year. The reason for this temporary increase is not clear. The mean number of inseminations that were applied per estrus (Fig. 5) remained more or less the same over time in farm B whereas on farm C, the number decreased from approximately 1.6–1.2 during the last quarters of the study. A lower number of inseminations combined with similar or even better reproductive performance is economically beneficial for the farm.

The WEI, ED and the number of inseminations per estrus were also investigated in relation to the parity of the sows (Table 7). In both farms, the WEI was generally longer in young sows compared to older sows, although the difference was only significant in farm B. However, the parity effect on ED was different in the farms. The ED was significantly shorter in parity 1 sows in farm B, whereas the ED was significantly longer in young sows in farm C. A precise explanation is not clear. In literature, ED is considered to be shorter in young sows than in older sows [2]. Kemp and Soede [7] reported ED to be between 46 and 53 h for sows and 36–48 h for gilts. The results on parity effects indicate that it is important for the farmer to be aware of the differences of WEI and ED between different parities in order to optimize timing of insemination. Next to that, in both farms, the number of inseminations per estrus was significantly higher in younger compared to older sows. This might suggest that the system scores better when sows get older, possibly due to less behavioral variation in older sows.

This study included three Belgian farms that were selected based on their willingness and motivation to use the SSB system. As the farms were not selected randomly, the results cannot be extrapolated to the entire Belgian sow population. Nevertheless, the three different farms are similar to many other pig farms in Belgium and Europe in terms of management practices, housing conditions, nutrition, genetics and reproductive performance. Also, the farms were monitored for quite a long period of time namely 1.5 year before and 1.5 year after implementing the SSB system, covering all seasons of the year, and including more than 6700 reproductive cycles. As the SSB system was used on all the sows in the farm, it was not possible to have a simultaneous control group but we had to work with a historic control group. It is known that next to a proper estrus detection, many other factors such as management, genetics, feed, health status and sperm quality are of major importance to increase the chance for a successful conception [3].

Those factors may change over time and thus might have influenced in some way the results e.g. increasing litter sizes as a result of genetic improvement [15]. However, interviews with the farmers pointed out that no major changes regarding these factors took place during the study period, except for the estrus detection by the SSB, and associated with it, the new pipettes and the insemination dose in farms B and C.

## Conclusion

This study showed that an AI system for estrus detection in sows, when used properly, can help farmers to improve the reproductive performance, assess estrus characteristics and reduce the number of inseminations per estrus. The results varied per farm, implying that other factors such as management, genetics and insemination dose also influence reproductive performance. It was also shown that the AI system detected estrus earlier after weaning and with less variation in comparison with estrus detection by the farmer. Further research in more farms is warranted, also to assess the economic benefits of using such AI systems in sows.

## Data Availability

The datasets used and/or analyzed during the current study are available from the corresponding author upon reasonable request.
